# GnRH - a *Missing Link* between Testosterone Concentrations in Yolk and Plasma and Its Intergenerational Effects

**DOI:** 10.1371/journal.pone.0022675

**Published:** 2011-07-28

**Authors:** Wendt Müller, Ton G. G. Groothuis, Vivian C. Goerlich, Marcel Eens

**Affiliations:** 1 Department of Biology-Ethology, University of Antwerp, Antwerp, Belgium; 2 Behavioural Biology, University of Groningen, Groningen, The Netherlands; University of Lethbridge, Canada

## Abstract

Despite the strong interest in hormone-mediated maternal effects two key questions concerning their mechanisms are as yet unanswered: First, whether the deposition of hormones in the egg yolk is coupled with the levels of these hormones in the maternal circulation, and second, whether epigenetic changes as induced by embryonic exposure to maternal yolk hormones impinge on yolk hormone deposition at adulthood. We investigated the responsiveness to gonadotropin-releasing hormone (GnRH) in female canaries whose embryonic exposure to yolk testosterone had been manipulated. This enabled us to study to what extent GnRH interlinks testosterone concentrations in female circulation and egg yolk as well as the intergenerational potential of hormone-mediated maternal effects. As expected, canary females responded to GnRH with a rise in plasma testosterone. The GnRH-responsiveness was positively correlated with the yolk testosterone content. Factors stimulating the release of GnRH will, therefore, lead to an increase of testosterone in both plasma and egg, posing a potential constraint on the yolk hormone deposition due to testosterone related trade-offs within the laying female. Exposure to elevated yolk testosterone levels as embryo reduced the GnRH-responsiveness in adulthood, potentially limiting environmental influences on yolk testosterone deposition, but the concentrations of yolk testosterone itself were not affected.

## Introduction

Maternal effects are defined as phenotypic variation in offspring that is a consequence of the mother's phenotype rather than the genetic constitution of the offspring [1]. Maternal effects can have a substantial influence on evolutionary processes due to their enormous potential to create immediate phenotypic responses in offspring via developmental plasticity [2]. The role of maternal effects in ecology and evolution has, therefore, received increased attention during the past decade - also stimulated by the landmark publication *Maternal effects as adaptations* by Mousseau and Fox [3].

In birds and other oviparous vertebrates one specific type of maternal effects, so-called hormone-mediated maternal effects, where offspring phenotype is influenced by maternally derived yolk hormones, has received considerable interest in behavioral and evolutionary ecology [4] (reviewed in [5], [6]). This flourishing field succeeded in advancing our knowledge of the functional and evolutionary significance of maternally derived yolk hormones. However, proximate questions concerning the mechanisms shaping hormone-mediated maternal effects have largely been neglected. This unbalanced knowledge may hamper further progress as the costs and benefits of hormone-mediated maternal effects also depend on the mechanisms that are available to both females, when depositing hormones in their eggs, and offspring, when responding to maternal yolk hormones during early development [7], [8]. The necessity of studies aiming at the physiological mechanisms has repeatedly been highlighted in several recent reviews dealing with hormone-mediated maternal effects [8]–[10]. This study investigates proximate aspects, focusing in particular on the mechanisms of yolk hormone deposition and the potential for transgenerational effects.

The enormous variation in hormone deposition that has been observed in relation to environmental factors (reviewed in [5], [6]) may be taken as evidence for a high degree of phenotypic plasticity in hormone deposition. However, the fact that the same environmental factors that modulate yolk hormone concentrations also affect plasma hormone levels in females raises the question whether and to what extent the transfer of hormones to the egg is regulated independently from the regulation of circulating hormone levels in the mother [6], [8]. This is currently one of the most central questions for our understanding of the potential trade-offs underlying yolk hormone deposition since a link between maternal and egg hormone levels would force the females to trade off the effects of the hormone on herself against those on her offspring. Previous studies investigated this link between hormones in plasma and yolk by implanting or injecting females with hormones, showing an increase in yolk hormone concentrations following manipulation [11]–[14]. However, the primary source of the gonadal hormones are the cell layers of the follicular wall surrounding each growing oocyte [15], so that hormones produced here are likely to enter the ovum without being first transferred to the circulation [8]. The more appropriate way to test for a link between hormones in plasma and yolk is to stimulate the ovary to produce hormones itself by elevating the levels of luteinizing (LH) or follicle-stimulating (FSH) hormones that stimulate hormone production in the ovaries or by the gonadotropin-releasing hormone (GnRH) that in turn stimulates LH or FSH release, which then again stimulate the theca interna cells (and to a lesser extent the granulosa cells) of the ovarian follicles to synthesize androgens, which can then again be released [16], [17] (reviewed in [15]). We focus on the latter since it has recently been linked to yolk testosterone deposition. Jawor and colleagues [18] reported that in females with developing follicles, the responsiveness of the HPG axis to a challenge with GnRH as measured as increase of plasma testosterone concentration in the female circulation correlates positively with the testosterone concentration of the yolk. This suggests that factors stimulating the release of GnRH during egg formation may result in higher levels of yolk testosterone and at the same time higher levels of plasma testosterone, which in turn may influence female behaviour and physiology. Because of the latter we also measured the reproductive performance, which is known to be modulated by testosterone (e.g. [19]).

In addition, we investigate the possibility that yolk hormone deposition is the target of trans-generational priming. Embryonic exposure to maternal yolk hormones has long-lasting phenotypic consequences (reviewed in [5], [6]), and it is likely that these phenotypic changes concur with yet unknown modifications of the endocrinological system [8]. These changes in endocrinology may include mechanisms important for yolk hormone deposition such as the responsiveness of the HPG axis - creating substantial potential for the transmission of phenotypic changes throughout generations. In addition, long-lasting phenotypic changes due to elevated exposure to maternal androgens may not only have consequences for yolk hormone deposition, or morphological and behavioral traits [20]–[22], but may affect female reproduction in general, as a number of female reproductive traits have been shown to be negatively affected by testosterone [19].

We, therefore, performed our study with female canaries (*Serinus canaria*) who were exposed to experimentally manipulated levels of yolk testosterone (increased versus sham-treated) during embryonic development [22]. We hypothesized that females hatched from an egg with elevated yolk testosterone levels would show a higher responsiveness to GnRH, make a smaller reproductive investment and lay eggs with higher yolk testosterone levels themselves, as we expected to find a positive correlation between the responsiveness to GnRH and yolk testosterone deposition.

## Materials and Methods

### Ethics statement

The study was performed under proper legislation of the Belgian and Flemish law and was approved by the ethical committee of the University of Antwerp (ID 06/19).

### (a) Origin of the experimental birds

The canary females (*Fife Fancy* strain) originate from an experiment testing context dependent effects of yolk testosterone on early development (for more details see [22]). Briefly, we manipulated yolk testosterone concentrations of the first and second laid egg by injecting either 50 ng testosterone dissolved in 5 µl sesame oil (in order to elevate the concentrations of the first laid eggs to the levels of later laid eggs as in canaries yolk T concentrations increase over the laying order, [22]) or 5 µl sesame oil only as control. In addition, we experimentally controlled the size asymmetry in order to mimic hatching asynchrony, placing two heavier and older chicks (*seniors*, one chick hatching from a testosterone treated egg and one chick hatching from a control treated egg) together with two younger and lighter chicks (*juniors*, again each one chick of both treatments) in an experimental nest. However, belonging to the *senior* or *junior* category did not have a significant effect in any of the analyses in this study (see below).

After independence at about 30 days of age, we kept all birds in large indoor aviaries separated for sex but mixed for treatment until the start of this experiment in spring 2009. We then selected all unrelated females (N = 43) for this experiment. 33 out of 43 birds were raised in the design as described above. In addition we used 11 females hatched from control- or testosterone-treated eggs that could not be cross-fostered according to the experimental scheme. These females were raised by foster parents (brood size 2–4) with a varying degree of size and age asynchrony. Females hatched from control treated eggs will henceforth be referred to as C-females, females hatched from testosterone treated eggs as T-females. The experiments started nine weeks after the light regime was changed to 14∶10 L∶D (the beginning of February).

### (b) GnRH challenge and egg collection

All females were mated with unrelated 2-year old males from the local breeding population of canaries. All pairs were housed in separate breeding cages equipped with nest boxes and nesting material. Throughout the experiment, we provided the birds with canary seed mixture (van Camp, Belgium), water, shell grit, and cuttlefish bone ad libitum and twice weekly with egg food (van Camp, Belgium).

We aimed to measure the female responsiveness to GnRH during the time that her eggs were developing, which appears to be the only period in which females are responsive to GnRH [18]. Therefore, we measured the responsiveness to GnRH on the day that the nest was nearly completed, which is in canaries on average about 5 days prior to egg laying. Females were taken from their cage and an initial blood sample (∼100 µl) was taken immediately. Thereafter, we injected 1.25 µg c-GnRH-I dissolved in 50 µl phosphate-based saline into the *pectoralis major* muscle (concentrations are based on the study by Jawor et al. [18]). All females were injected early in the morning starting at about 9.00 a.m., and the order of T- and C-females was alternated whenever possible. The females were placed into small boxes until the second final blood sample (∼100 µl) was taken 30 min after injection, which is the assumed time point of peak response [23] (see also [24], [25]). We strictly followed this time protocol in order to standardize potential effects of stress on plasma testosterone levels [25], [26]. Subsequently, the females were returned to their cage and the blood samples were centrifuged. We separated the plasma and immediately froze it at −20°C until analysis.

Nests were checked daily and freshly laid eggs were marked and replaced by dummy eggs. The eggs were weighed (to the nearest 0.001 g) and immediately frozen at −20°C until further analysis. The experiment was terminated two days after the last egg had been laid and the birds were returned to their aviaries.

### (c) Hormone analysis

Testosterone concentrations of the initial and final plasma samples, as well as in the yolks of first laid eggs were determined based on a previously established and validated protocol [27]. The whole yolk was removed from the frozen egg, weighed to the nearest 0.001 g, diluted with distilled water (1 ml water per gram of yolk), and homogenized on a vortex. The whole plasma sample or circa 100 mg yolk mixture, respectively, was extracted by adding 2.5 ml of diethyl ether/petroleum benzene, 70∶30 (vol/vol). The mixture was vortexed, centrifuged, and after snap freezing the organic phase was decanted into fresh tubes. The extract was dried under a stream of nitrogen and the procedure was repeated once. A single extraction with 1 ml of 70% methanol followed. After overnight freezing at −20°C the samples were again centrifuged, decanted and dried. Plasma samples were re-suspended in 110 µl phosphate buffered saline (PBS), yolk samples in 400 µl. We measured testosterone concentrations using commercial radioimmunoassay (RIA) kits (Actives- Testosterone Coated-Tube RIA DSL-4000 kit; Diagnostic Systems Laboratories, Beckman Coulter Nederland B.V., Woerden, The Netherlands) with a detection limit of 0.02 ng/ml. The kit antibody cross-reacts 100% with testosterone, 5.8% with 5a- dihydrotestosterone, and 2.3% with androstenedione. All plasma samples and yolk samples were measured in one assay each. The intra-assay coefficient of variation was 3.05 (plasma) and 3.09 (yolk).

### (d) Statistical analyses

Data were checked for normality and homogeneity of variances, if necessary transformed and all analyses were performed in SPSS 14.0. Data are shown as mean ± se of the mean unless stated otherwise. The plasma testosterone concentration of one initial (7.00 ng/ml) and one final sample (7.72 ng/ml) of in total two females (one C- and one T-female) were outliers and excluded from the statistical analyses.

For the subset of birds (33 out of 43), which were cross-fostered according to the experimental scheme of our previous study [22], we also included in the analysis whether a female was *senior* (heavier and older chick) or *junior* (younger and lighter chick) sibling as a categorical variable. Belonging to the *senior* or *junior* category did not have a significant effect in any of the analyses neither in itself nor in interaction with the yolk testosterone treatment. The respective detailed statistics are, therefore, not explicitly stated in the results section.

## Results

### (a) Plasma T levels, responsiveness to GnRH and yolk T deposition

The initial plasma testosterone concentrations decreased with an increasing time difference between sampling and laying of the first egg { = SL-interval, please note that the SL-interval is shorter than the laying latency (see below) as females were not necessarily injected on the day of pair formation} (linear regression, t = −2.73, p = 0.009, N = 42), indicating that T levels increased when closer in time to egg laying. We, therefore, used the residuals of the initial plasma T levels on the SL-interval to correct for the length of the SL-interval when analyzing the relationship of the plasma T-levels with yolk hormone deposition. The residuals of the initial plasma concentration did not correlate with the yolk testosterone concentrations (Pearson's r = −0.04, p = 0.81, N = 42) or the total amount of testosterone deposited in the yolk (Pearson's r = −0.005, p = 0.97, N = 42).

**Figure 1:**
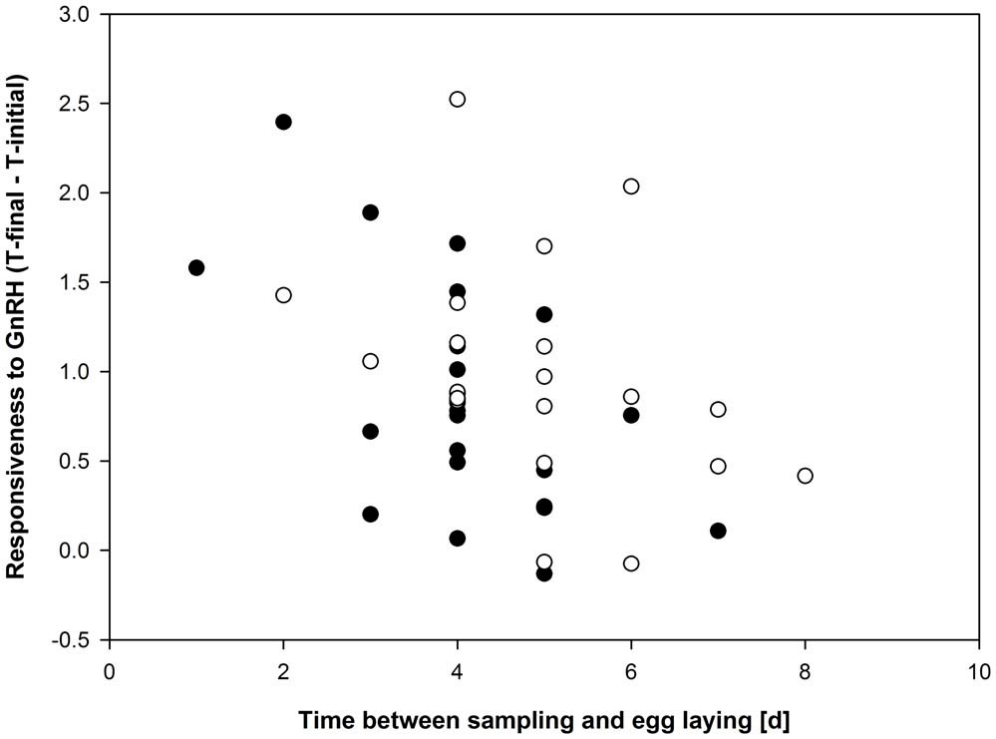
Increase in plasma testosterone concentrations following a challenge with gonadotropin-releasing hormone (GnRH). Increase in plasma testosterone concentrations (ng/ml) 30 min after injection of GnRH in relation to the laying stage. Data are split for females hatched from control-treated eggs (C-females, open circles) and females hatched from testosterone treated eggs (T-females, filled symbols).

**Figure 2:**
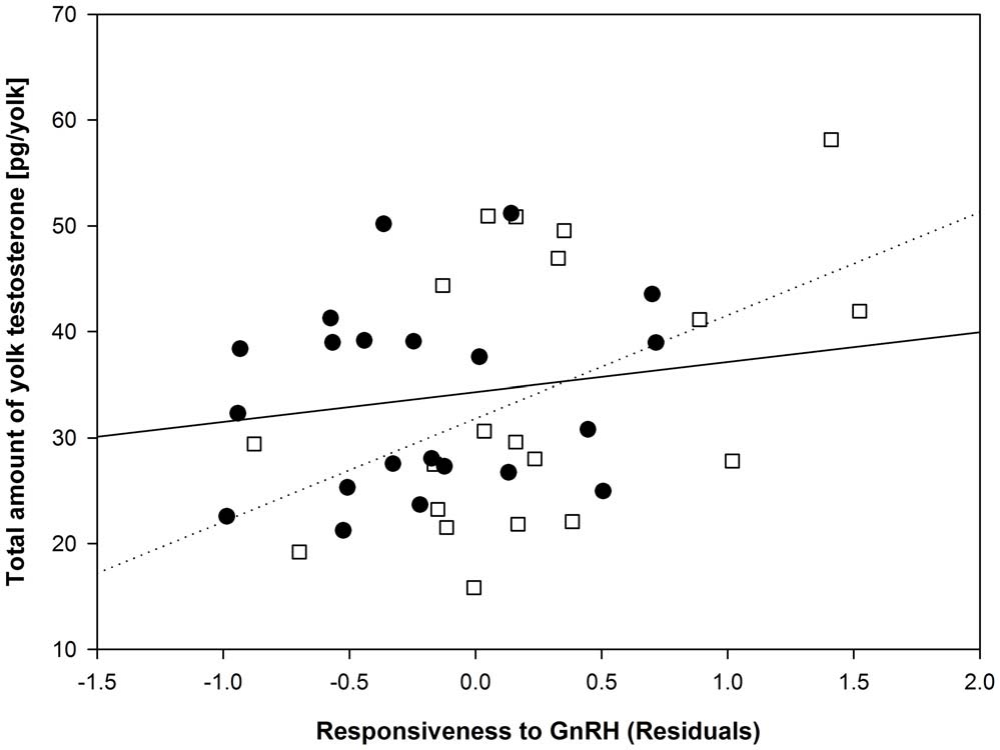
Relationship between testosterone response to a challenge with GnRH and yolk testosterone. Relationship between the increase in plasma testosterone concentrations 30 min after injection of GnRH and the amount of testosterone deposited in the yolk, using the residuals of the responsiveness to GnRH on the length of the time interval between injection of GnRH and laying of the first egg. Data are split for females hatched from control-treated eggs (C-females, open symbols and dotted line) and females hatched from testosterone treated eggs (T-females, filled symbols and solid line).

The responsiveness to GnRH, which is defined as the post-challenge increase in plasma testosterone concentrations (final plasma testosterone concentrations – initial plasma testosterone concentrations) decreased with an increasing length of the SL-interval (linear regression, t = −2.78, N = 41, p = 0.008) (Figure 1). There was no correlation between initial plasma T levels and the responsiveness to GnRH (Pearson's r = 0.10, p = 0.55). We then analyzed whether the responsiveness to GnRH correlated with the deposition of yolk testosterone, using the residuals of the responsiveness to GnRH on the length of the SL-interval, since GnRH sensitivity increases when the time of laying the first egg comes closer and the latter varied among females. There was no such correlation with yolk testosterone concentrations (Pearson's r = 0.23, p = 0.14, N = 41), but a significant positive correlation with the total amount of yolk testosterone (Pearson's r = 0.33, p = 0.04, N = 41) (Figure 2).

### (b) Intergenerational effects of embryonic testosterone exposure

There were no differences in the initial plasma testosterone concentrations between C- (median 0.62, 25^th^ percentile 0.36, 75^th^ percentile 0.78 ng/ml) and T-females (median 0.54, 25^th^ percentile 0.47, 75^th^ percentile 1.14 ng/ml) (ln-transformed, F_1,39_ = 0.30, p = 0.59, SL-interval included as covariate). However, T-females (0.76±0.13 ng/ml) were less responsive to GnRH compared to C-females (1.08±0.16 ng/ml) (GLM, F_1,38_ = 6.33, p = 0.02, SL-interval included as covariate)(Figure 1). Given the difference in responsiveness to GnRH between C- and T-females, we re-analysed the relationship between the responsiveness to GnRH and the yolk testosterone deposition (see above). Interestingly, the correlation between responsiveness to GnRH (using the residuals of the responsiveness to GnRH on the length of the SL-interval) and the total amount of T deposited in the yolk remained significant for C-females (Pearson's r = 0.46, p = 0.04, N = 20), but not for T-females (Pearson's r = 0.16, p = 0.49, N = 21). However, the correlations are not significantly different (z = 1.01, p = 0.15) and do not differ in their slopes (t = 1.19, p = 0.24).

This pattern is similar when analysing the yolk testosterone concentrations, with the correlation in C-females now approaching statistical significance (C-females: Pearson's r = 0.41, p = 0.07, N = 20; T-females Pearson's r = 0.11, p = 0.65, N = 21). Both correlations are not significantly different (z = −0.62, p = 0.27) and there is no slope difference (t = 1.11, p = 0.27).

In order to facilitate a comparison with the Jawor et al. study [18], we repeated the analysis for C-females without correction for the SL-interval. Interestingly, both correlations appear to be significant as in the Jawor et al. study [18] (yolk testosterone concentrations: Pearson's r = 0.45, p<0.05; total amount of yolk testosterone: Pearson's r = 0.45, p = 0.04). The fact that the relationship between responsiveness to GnRH and yolk testosterone concentrations, slightly improves in C-females if we do not take the SL-interval into account likely relates to the fact that the yolk mass increases with a longer SL-interval (linear regression, t = 2.20, N = 20, p = 0.04).

### (c) Egg mass, yolk mass and yolk testosterone

C- and T -females did not differ in body mass at pair formation (F_1,41_ = 0.40, p = 0.53). T-females initiated egg laying (*laying latency*) earlier than C-females (T-females: median 7, between 5–12 days; C-females: median 7, between 6–21 days, Mann-Whitney U test, Z = −2.18, p = 0.03, N = 43) (Table 1). But there were no differences between C- and T-females in clutch size (Mann-Whitney U test, Z = −1.37, p = 0.17, N = 43)(Table 1), egg mass (F_1,41_ = 0.44, p = 0.51) (Table 1) or clutch mass (F_1,41_ = 2.15, p = 0.15) (Table 1). However, eggs of T-females contained significantly smaller yolks compared to C-females (F_1,40_ = 4.77, p = 0.04, SL-interval included as covariate F_1,40_ = 4.90, p = 0.03) (Table 1). Eggs of C-females and T-females did not differ in their yolk testosterone concentrations (F_1,41_ = 0.47, p = 0.50) (Table 1) or the total amount of testosterone deposited (F_1,41_ = 0.01, p = 0.90) (Table 1).

**Table 1:** Female reproduction in relation to yolk testosterone, plasma testosterone and testosterone response to GnRH.

	C-females			T-females				R-Initial		R-GnRH	
	median	25th	75th	median	25th	75th	p-value	r/r_s_	p-value	r/r_s_	p-value
Time till first egg [d]	7	7	8.5	7	6	7	*0.03*	−0.2	0.2	0.14	0.4
Clutch size	4	4	5	5	4	5	0.17	0.01	0.77	−0.45	*0.003*

	mean	s.e.		mean	s.e.						
Average egg mass [g]	1.78	0.03		1.81	0.03		0.51	0.18	0.25	0.14	0.38
Clutch mass [g]	7.42	0.31		7.81	0.28		0.15	0.18	0.26	−0.39	*0.01*
Yolk mass (first egg) [g]	0.32	0.01		0.3	0.01		*0.04*	0.02	0.92	0.14	0.4
Yolk T concentration [pg/mg]	107.42	9.83		115.62	6.92		0.50	−0.04	0.81	0.23	0.14
Yolk T content [ng/yolk]	33.96	2.7		33.64	1.85		0.90	−0.01	0.97	0.33	*0.04*

Summary of the reproductive traits measured according to yolk hormone treatment (mean ± s.e. or median ±25^th^/75^th^ percentile in case of not normally distributed data) and their relationships with the plasma testosterone concentrations of the initial blood sample and the increase in plasma testosterone concentrations in response to a challenge with GnRH, using the residuals of the plasma testosterone concentrations (R-initial) respectively the responsiveness to GnRH (R-GnRH) on the length of the time interval between injection of GnRH and laying of the first egg for the analysis (Pearson's r or Spearman's r_s_ in case of not normally distributed data).

We explored the potential relationships between the endocrine parameters of the female and her reproductive performance. However, there were no correlations between the maternal plasma testosterone concentrations and the reproductive traits (using the residuals of the initial plasma testosterone levels on the length of the SL-interval) (Table 1). The residual responsiveness to GnRH (residuals of the responsiveness to GnRH on the length of the SL-interval) was negatively correlated with clutch size and clutch mass (Table 1).

## Discussion

Maternal hormones in bird eggs have recently received much attention, since they represent an intriguing pathway for *maternal effects*. However, the underlying mechanisms remain largely elusive. By means of manipulation of testosterone production in females via the relevant physiological pathway (injection of GnRH), we studied the mechanism of testosterone deposition in the yolk, potential physiological trade-offs and, by using females from control and testosterone injected eggs, intergenerational effects as well as their pathways. These topics will be discussed subsequently.

### Mechanisms of yolk testosterone deposition

Female canaries responded to a challenge with GnRH during the egg development phase with an increase in plasma testosterone concentrations. This confirms that females are indeed responsive to GnRH during this specific period and respond to GnRH via the steroid production of the ovarian follicles [18] (see also [24]). The type and amount of hormones produced varies with the developmental stage of the follicle [28], [29] (reviewed in [15]), and depends, therefore, both on their number and their developmental stage. In passerines, the total steroidogenic output increases during egg formation [30], [31], which may explain why both, initial plasma concentrations and response to GnRH increase closer to egg laying, as more follicles are maturing [32]. When analyzing the relationship between responsiveness to GnRH and yolk testosterone concentrations (and all subsequent analyses) we therefore corrected for this time effect and future studies aiming at measuring the responsiveness to GnRH should certainly take this fine scale effect into account.

The (residuals of the) responsiveness to GnRH were positively correlated with the yolk testosterone content of the first egg. Thus GnRH forms an important link between maternal hormone deposition and maternal plasma concentrations with significant consequences for physiological trade-offs within the laying female. However, the relationship between the responsiveness to GnRH and yolk testosterone concentrations was less strong than in the study by Jawor and colleagues [18]. This is likely due to the fact that this relationship is modulated by embryonic testosterone exposure, which was experimentally manipulated in this study (see below). Furthermore, the increase in yolk mass with an increasing time interval between GnRH challenge and yolk testosterone concentrations blurs the relationship between these traits. However, it does not challenge the GnRH dependent link between testosterone in yolk and plasma. It rather shows that yolk testosterone concentrations can be affected by the amount of hormones and/or the amount of yolk, which has to be taken into account when interpreting yolk testosterone concentrations.

Interestingly, it has recently been shown that the increase in circulating testosterone after a challenge with GnRH strongly correlates with the rise in plasma testosterone concentrations following a simulated territorial intrusion (STI) in males [33]. This may explain why aggressive interactions during egg formation lead to elevated yolk testosterone concentrations [34], [35]. If the increase of circulating testosterone, which enables the female to respond to a challenge, results from a stimulation of the ovarian follicles (via LH), it will at the same time lead to elevated yolk testosterone concentrations, since the accumulation of hormones in the yolk is due to the hormone secretion by the ovarian follicles. Thus, the source of testosterone in both plasma and yolk following a social challenge may be the same. An increase in yolk testosterone concentrations as a consequence of aggressive interactions does, therefore, not require that hormones will be passed from the maternal circulation to the yolk, as has often been assumed.

This dual role of GnRH in regulating yolk testosterone deposition and the levels of these hormones in the maternal circulation [18] indicates that factors stimulating the release of GnRH lead to higher levels of yolk hormones influencing chick development [5] while at the same time influencing female behaviour and other important – testosterone dependent – life-history traits. In particular this may have significant consequences for female reproductive performance, which is known to be negatively affected by testosterone [19]. Indeed, we found that females with a higher responsiveness to GnRH laid smaller clutches. Interestingly, such a pattern has also been shown for females implanted with testosterone [11], [12], [36] (but see [13], [37]). However, this has to be interpreted with caution, since the injection with GnRH might have had a direct effect on clutch size. Although the plasma testosterone concentrations were probably only elevated for a very short term, it might have had a negative effect on follicle development/recruitment. The rise in plasma testosterone levels, and thus the potential negative effect on clutch size, was stronger when the females were challenged closer to egg laying.

We did not find any significant relationship between (plasma/baseline) testosterone concentrations and reproductive performance. However, plasma testosterone levels are very dynamic especially during follicle development and may be a poor indication of the general endocrine state of an individual [38]–[40].

### Trans-generational priming of enhanced embryonic yolk testosterone exposure

Embryonic exposure to maternal yolk hormones can have long-term phenotypic consequences eventually lasting until the age of reproduction (reviewed in [5], [6]; for this species see [22]). In females, elevated yolk androgen levels affect, among other, behavioral traits such as aggressiveness (e.g. [21], [22]), which in turn may influence the amount of yolk androgens they will deposit in their eggs [34], [35]. However, here we clearly show that T-females ( = females hatched from testosterone treated eggs) do not differ in the amount or concentration of yolk testosterone from C-females ( = females hatched from control treated eggs), despite existing behavioral differences [22]. The results are in line with the only comparable study, performed under rather artificial circumstances, showing that an experimental yolk testosterone manipulation had no effect on the yolk testosterone concentrations of the eggs laid by these females at adulthood [41]. The fact that T-females did lay eggs with smaller yolks than C-females is again similar to the results of the previous study [41]. However, we did not measure offspring sex and it is, therefore, not possible to see whether this effect was restricted to eggs carrying a female embryo as in the previous study [41]. Further negative effects of embryonic exposure to elevated levels of yolk androgens have not yet been found in passerines [42]–[44] (see also [45]).

The embryonic exposure to elevated yolk testosterone levels also did not affect the plasma testosterone concentrations at adulthood [46]. Positive effects of elevated yolk androgen levels on endogenous testosterone production have to date only been reported during the early developmental period [47] (see also [48]). However, as pointed out above, the significance of single testosterone measurements at adulthood may be limited [38]–[40], as also indicated by the low within-individual repeatability of plasma testosterone concentrations in the study by Partecke and Schwabl [46].

Interestingly, we found that an embryonic exposure to elevated yolk testosterone levels did have a significant negative effect on the responsiveness to GnRH. This contrasts our expectations, given the positive effects of yolk androgens on the expression of androgen-dependent female plumage traits and behaviour [21] and on the ability to defend a food resource in females [20], [22]. However, here we show, like Jawor et al. [18] that the response to GnRH is probably dependent on the testosterone production of the pre-ovulatory follicles in the ovary, but none of the above mentioned studies have been performed during the period of egg development. Another reason why we expected positive effects of in ovo testosterone treatment are due to studies in mammals showing that excess prenatal testosterone exposure leads to an enhanced sensitivity of the pituitary to GnRH and hypersecretion of LH (e.g. [49]). One explanation for this apparent difference may be found in the comparatively small in ovo testosterone elevation in birds when compared to the testosterone treatment or its timing in mammals. However, in ovo exposure to glucocorticoid hormones has been shown to cause hyperactivity of the hypothalamo-pituitary-adrenal axis in both birds [50] and mammals (see [51] for a review).

Our study does not capture the full complexity of the endocrine processes that ultimately lead to elevated testosterone following GnRH injection. Future studies are, therefore, needed in order to understand the complex endocrine mechanisms underlying the differential production of testosterone between T- and C-females in response to GnRH injection in more detail. A prime target for these studies is certainly luteinizing hormone (LH), which is the primary hormone released after GnRH injection, and which is also strongly involved in the regulation of steroid production by the theca and granulosa cells [15]. Furthermore, the duration and shape of an endocrine response is an often neglected source of individual variation [40], and it is, therefore, also of interest to measure the time course of a response to GnRH depending on the embryonic exposure to yolk testosterone.

Embryonic exposure to yolk testosterone seems to weaken the relationship between the responsiveness to GnRH and the yolk testosterone deposition at adulthood, potentially limiting environmental influences on yolk androgen deposition via the mother, and thus potentially adaptive maternal effects [5]. Although this did not result in differences in yolk testosterone concentrations, it may become important in a socially more challenging environment, in which GnRH is frequently elevated, but at present this remains speculative. The responsiveness to GnRH was negatively correlated with clutch size, which should have led to larger clutch sizes in T-females. The fact that this difference did not reach statistical significance indicates that this effect may be rather small. But as pointed out above, this has to be interpreted with caution as the GnRH injection might have had an effect on clutch size.

Thus the changes in responsiveness to GnRH are as yet insufficient to enable us to understand the long-lasting consequences of embryonic exposure to yolk testosterone. Embryonic exposure to yolk androgens may in addition sensitize certain neural circuits in the brain, induce changes in receptor sensitivity/density, and/or cause epigenetic changes with differences to be found on the gene expression level, but it will require additional studies to answer these questions.
